# COVID-19 Mixed Impact on Hospital Antimicrobial Stewardship Activities: A Qualitative Study in UK-Based Hospitals

**DOI:** 10.3390/antibiotics11111600

**Published:** 2022-11-11

**Authors:** Sidra Khan, Stuart E. Bond, Mina Bakhit, Syed Shahzad Hasan, Ahmed A. Sadeq, Barbara R. Conway, Mamoon A. Aldeyab

**Affiliations:** 1 Department of Pharmacy, School of Applied Sciences, University of Huddersfield, Huddersfield HD1 3DH, UK; 2 Department of Pharmacy, Mid Yorkshire Hospitals NHS Trust, Wakefield WF1 4DG, UK; 3 Institute for Evidence-Based Healthcare, Bond University, Gold Coast, QLD 4226, Australia; 4 Department of Pharmacy, Shaikh Shakhbout Medical City in Partnership with Mayo Clinic, Abu Dhabi P.O. Box 11001, United Arab Emirates; 5 Institute of Skin Integrity and Infection Prevention and Department of Pharmacy, School of Applied Sciences, University of Huddersfield, Huddersfield HD1 3DH, UK

**Keywords:** antibiotics, antimicrobial stewardship (AMS) activities, COVID-19 pandemic, clinical practice, qualitative study

## Abstract

Antimicrobial resistance (AMR) is a well-known global threat due to the subsequent increase in antimicrobial usage. Several antimicrobial stewardship (AMS) strategies have been implemented to curb irrational prescribing and reduce the AMR burden. However, since the beginning of the COVID-19 pandemic, it has enormously impacted the healthcare system and jeopardized public health, causing millions of deaths globally. Our semi-structured qualitative study aimed to explore the impact of COVID-19 on AMS activities in the UK hospitals. Seventeen interviews were conducted with health care professionals who were part of AMS teams (consultant medical microbiologists, infectious disease consultants, antimicrobial pharmacists). Interviews were audio-recorded and transcribed. An inductive thematic framework was adopted to analyse and create the themes. After agreement of the hierarchical framework definition, all transcripts were coded accordingly. Four main themes and 15 sub-themes were identified. These main themes were: (1) AMS activities or strategies before and during the pandemic; (2) challenges to implementing AMS activities before and during the pandemic; (3) information from public authorities on AMS during the pandemic; and (4) new AMS activities/strategies adopted during the pandemic. Staff vacancies, redeploying of AMS staff to other duties and meeting the burden related to the COVID-19 and lack of resources were the most frequently identified contributing factors to withheld AMS activities during the pandemic. However, modifications to the hybrid working environment, i.e., remote or flexible working, allowed for resumption of AMS activities including virtual ward rounds, virtual meetings and other activities. Further research needs to assess the impact of the hybrid delivery system on AMS activities.

## 1. Introduction

Since the beginning of the COVID-19 pandemic, massive disruptions to healthcare activities have led to increased morbidity and mortality worldwide [1]. Despite COVID-19 being a viral disease, a high percentage of COVID-19 patients were prescribed antibiotics [2]. Due to the lack of clear guidelines and irrespective of disease severity or low rate of proven bacterial co-infection and secondary infection, empirical antibiotic therapy was widespread during the initial stages of the pandemic [3,4,5,6,7]. Moreover, a meta-analysis that collected data from high-income countries (HICs) and low-middle income countries (LMIC) revealed antibiotic use in 68% of COVID-19 patients. Sub-group analysis found that 58% of patients were prescribed antibiotics in HICs and 89% in LMICs [8]. This raises concerns about the impact of COVID-19 on empirical antibiotic therapy, withdrawal of antimicrobial stewardship (AMS) activities, and enhanced risk of antimicrobial resistance (AMR) [1,9,10,11,12]. Several studies from secondary health care settings in the United Kingdom (UK) reported increased antibiotic consumption with no evidence of bacterial infection [13,14,15,16].

AMR results in increased morbidity, mortality, and cost of health care and threatens public health globally [17,18]. A key driver of AMR is antimicrobial consumption [19,20,21]. Various approaches have been adopted to minimize the inappropriate use of antimicrobials [22,23]. The World Health Organisation (WHO) introduced the Global Action Plan on Antimicrobial Resistance in 2015, outlining five key objectives to optimise the use of antimicrobials in human and animal health [24]. In 2007, the Infectious Diseases Society of America (IDSA) guidelines used the term “Antimicrobial stewardship (AMS) program” to combat AMR [25]. AMS programs aimed to promote the rational use of antibiotics, prevent the emergence of AMR, enhance patient safety and healthcare outcomes, and reduce healthcare costs [24,25]. In 2007, the UK government established a Specialist Advisory Committee on Antimicrobial Resistance and Healthcare-Associated Infections (ARHAI) to provide scientific and expert governance on minimizing healthcare-associated infections (HCAI), controlling AMR, and optimizing antimicrobial use in humans [26,27]. AMS programs in the UK have a crucial role in mitigating the emergence of AMR [28]. The COVID-19 pandemic has substantially impacted AMS programs and AMR [12,29].

Numerous factors contributed to the inappropriate use of antibiotics for COVID-19, including decreased footfall on ward or virtual ward rounds, staff vacancies, lack of knowledge about COVID-19, and lack of diagnostics in hospital settings. In addition, traditional AMS activities were not prioritized during COVID-19 because health care providers were too engaged in dealing with COVID-19 transmission and providing treatment and preventive measures.

The primary focused of our study was AMS activities and strategies in the secondary healthcare setting during the COVID-19 pandemic. Our qualitative study aimed to explore the views, perceptions, and challenges of AMS team members from various healthcare professions in the UK.

## 2. Results

Seventeen interviews were performed between February and April 2022, averaging 36 min (range 24–58 min; total 616 min). All participants recruited were from seventeen different secondary healthcare facilities in the UK. Participants’ characteristics are reported in the supplementary material. The majority of participants were aged between 40–49 (*n* = 12), AMS pharmacists (*n* = 9), and were located in England (*n* = 11). Table 1 presents the interview and study participants.

### Thematic Analysis

**Figure 1:**
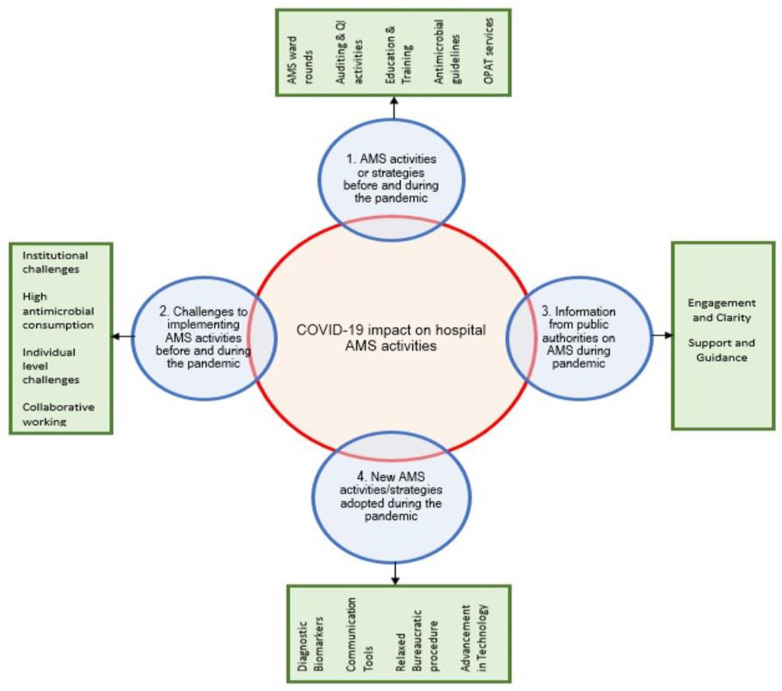
Themes (blue) and subthemes (green) emerged from the qualitative data.

From the data collected from the participants, four main themes and 15 sub-themes were identified, as shown in Figure 1. These main themes were: (1) AMS activities or strategies before and during the pandemic; (2) Challenges to implementing AMS activities before and during the pandemic; (3) Information from public authorities on AMS during a pandemic; and (4) New AMS activities/strategies adopted during the pandemic. An illustrative quote was recorded (Table 2, Table 3, Table 4 and Table 5), and a description of each subtheme was then recorded, summarising what was found.

## 3. THEME ONE: AMS Activities or Strategies before and during the Pandemic

### 3.1. AMS Ward Rounds

Participants mentioned antimicrobial stewardship (AMS) ward rounds as the most effective activity in implementing AMS strategies. Participants highlighted that having a good relationship with the staff was one of the main drivers of its effectiveness

*“… the most useful is the ward rounds that we do with the microbiologist, which has been really successful in terms of getting us actually exposure to staff across the trust. Because not just participate in the ward rounds but being seen and being active and answering queries helps to generate more queries.”*

(PI12)

Since the inception of the COVID-19 pandemic, AMS ward rounds have been neglected.

*“The thing that was probably impacted the most was the micro ward rounds because the microbiologists at that time we only had two, and they were so caught up in COVID planning that they didn’t have the time to do a ward round. So, they are really only prioritizing patients that would have been going or do they go home with antibiotics because the key was to get people out of the hospital. So those ward rounds were impacted the most.”*

(PI04)

### 3.2. Auditing and Quality Improvement Activities

Auditing and quality improvement was one of the most important AMS activities, which was also the most impacted activity during and before pandemic. A few participants discussed the Antibiotic Review Kit (ARK) [30,31] protocol which supports healthcare providers in switching antibiotics if they are no longer required [31].

*“Before the COVID pandemic… there was the ARK (Antibiotic Review Kit) study. I am not sure if you are familiar with it. We also took part in that again before the COVID pandemic, where we had automatic stop dates of antibiotic prescriptions after 72 h. So, if you wanted to prescribe more than 72 h of antibiotics, we had to… re-prescribe it on the Kardex and that influenced and increased our IV to oral switch rate that was again before the COVID pandemic.”*

(PI04)

Participants gave views on other helping tools for AMS activities: Start-smart-then-focus [32], and Commissioning for Quality and Innovation (CQUIN) [33] aiming to improve antimicrobial prescribing, which were also impacted during the pandemic. A participant also reported about Hospital Antibiotic Review Program (HARP) [34],

*“And that’s kind of where we were pre- COVID, the Scottish Antimicrobial prescribing group had just launched the HARP (Hospital Antibiotic Review Program) toolkit, and we were just starting to look at how we would use that as a tool and where we would employ it.”*

(PI01)

### 3.3. Education and Training

Participants highlighted that education and training are an integral part of AMS activities, which staff members positively received.

*“I think the things that were effective are education and training and so I think that’s always worked really well and we’ve always had very good feedback from junior doctors about that. And I feel like they sort of listen to what we recommend.”*

(PI05)

Before COVID, participants mentioned that educational seminars and workshops were conducted throughout the year to keep practitioners engaged in AMS activities. Most participants mentioned AMS education and teaching activities were ceased because of the COVID-19 pandemic,

*“I say that teaching a lot of it stopped, but it did present some additional options for teaching. So, for example, I was asked to give a teaching session to the acute medicine doctors about the use of procalcitonin testing. So yeah, some teaching was replaced by teaching that wouldn’t have otherwise the patterns under normal circumstances.”*

(PI17)

### 3.4. Antimicrobial Guidelines

Participants reported views regarding updating antimicrobial guidelines, using tools to access various antimicrobial guidelines through the MicroGuide-app [35], and introducing new guidelines during the pandemic.

*“And the other thing we do is, we have a micro guide electronic app… with all our antimicrobials policy on it and what we do is twice a year we do a snapshot point prevalence audit of the compliance with our micro guide… so we can see… how well everyone sticking to policy and see if there are any particular teams on the areas that… we need to work in.”*

(PI15)

### 3.5. Outpatient Parenteral Antimicrobial Therapy (OPAT)

Some participants reported that OPAT services, a cost-effect strategy to treat stable patients to complete antimicrobial courses as ambulatory outpatients or at home [36], were also affected by the pandemic.

*“I am sure the lack of availability we have had a big impact on our OPAT service. We had to pause our OPAT service during the pandemic again because we’re a smallish centre and we didn’t have the resource as well as running a COVID ward, we are now realizing how negative that was as we start restarted the service, and we are seeing patients who should have been treated appropriately, sometimes with longer courses of antibiotics and in fact, they were given up for specialist infections who… did not have as good outcomes as they might have had if we had a functioning well OPAT service and that access to specialist advice.”*

(PI01)

## 4. THEME TWO: Challenges to Implementing AMS Activities before and during the Pandemic

Participants highlighted challenges to implementing AMS either at the individual or organizational level. These challenges included meeting burdens, lack of resources or financing, engaging different stakeholders, staff vacancies, and obstacles in updating guidelines as major organizational-level challenges.

### 4.1. Institutional Challenges

Participants perceived an increase in the meeting burden during pandemic at different hierarchical levels

*“We had a massively increased meeting burden and increase in bureaucracy because we are constantly trying to write guidance and re-write guidance as the guidance changes for COVID and get back as quickly as possible so that we can help our clinical teams on the ground.”*

(PI06)

Participants mentioned challenges at the organizational level; lack of funds and staff vacancies were the major challenges.

### 4.2. High Antimicrobial Consumption

Most of the participants reported irrational antimicrobial prescribing, the reasons being that COVID-19 was a new disease, with insufficient information and guidelines and a lack of evidence-based practice; this caused increased trends of antimicrobial consumption during the initial phase of the pandemic.

*“… at the start of the pandemic, I would say no because we had quite an irrational use of antibiotics, it was given to everybody. However, as time moved on, we started to get the support of clinicians outside of the core antimicrobial stewardship team, such as the intensivist, such as the medical doctors who were covering, who ended up on the COVID rotations and they then started to highlight the use of inappropriate prescribing and that we need to curb the amount of antibiotics were using.”*

(PI08)

*“I think in particular for COVID-19 patients, there has been an overuse of antibiotics because of a lack of familiarity with the condition you are around, the rates of co-infection, etc. But I think even though when the evidence has come through, that hasn’t resulted in big changes in practice, and there is a lot of empirical antibiotic use that will be unnecessary in those patients. And I think other than that; we did refrain within our organization from changing too much around the antibiotic guidelines.”*

(PI11)

### 4.3. Individual-Level Challenges

During the pandemic, AMS staff also encountered challenges at the individual level in building relationships and collaborative working.

*“I think there were difficulties in those relationships and trying to get the respiratory team on board with helping to limit irrational use. So, in that sense I think in terms of the support of the different teams, it was building that relationship where you’re both on the same agenda and you both and I agree on what needs to be done. And there were many respiratory consultants eager to still prescribed antibiotics even if there was lack of evidence of infection.”*

(PI10)

### 4.4. Collaborative Working

Participants discussed building relationships with colleagues and the involvement of the clinical team in AMS activities

*“I think the key to running AMS programs in any department a relationship building. So, you know, finding interested champions of AMS within medical department, haematology department or ICU department and it’s down to us as microbiologist or infection doctors to cultivate these relationships. And only if we have these relationships with the department that we want to change is there any hope of changing.”*

(PI02, IDM)

*“Obviously I would prefer the clinical team to review the antibiotics daily and stop it accordingly in their ward round because obviously there is only two consultants working in this hospital. And yes, I do get the support because if I stop the antibiotics myself and most of the time, they do kind of accept that…”*

(PI09, MB)

## 5. THEME THREE: Information from Public Authorities on AMS during Pandemic

Participants perceived a lack of support and guidance from public authorities during the pandemic:

### 5.1. Engagement and Clarity

Participants showed concerns about the lack of clarity and engagement from public health authorities

*“I think that the health boards drive the agenda rather than public health… doing so. And often, its actions and initiatives that we have done back in the health boards that we feed up to our own delivery board that then changes national strategy. I don’t believe that public health has a good buy-in, I don’t believe that they provide good data, and I don’t believe that they give us good information on prescribing rates or anything out from that side at all. Most of it is done in-house and therefore, we are not all on the same strategic pace and then strategic page either.”*

(PI08)

### 5.2. Support and Guidance

Participants also raised concerns about the lack of guidance and information regarding AMS and COVID in general

*“we didn’t receive any information from our local public health agency on antibiotic stewardship during COVID… And each trust has taken a different approach and we’ve all kind of just gone and done our own thing on what we think, and you know, we should be doing. So, that’s my opinion. I don’t think we had any guidance from public health agency on what we should be doing regarding after antimicrobial stewardship.”*

(PI04, PH)

## 6. THEME FOUR: New AMS Activities/Strategies Adopted during the Pandemic

During the first year of the pandemic, most of the AMS activities were neglected; however, participants reported resuming routine activities at some level. Due to modifications to the hybrid-working environment and introduction resumed. Furthermore, various communication tools such as Microsoft teams, ZOOM, and Webex are widely used new delivery modules in the healthcare system to resume activities remotely, including virtual AMS word rounds; virtual AMS audits and virtual AMS meetings were commonly observed activities.

### 6.1. Diagnostic Biomarkers

Almost all participants gave encouraging views on introducing procalcitonin testing [37] to curb inappropriate antimicrobial use.

*“There has been an increased use in PCT or procalcitonin tests to help guide or monitor infection and know if antibiotic treatment is necessary or not. And that’s been quite widely adopted, and I think that’s has been a good intervention in terms of antimicrobial stewardship activity and of implementing.”*

(PI10)

Two participants mentioned introducing antiviral protocols as AMS activity and establishing new outpatient COVID centres for reviewing antivirals.

### 6.2. Communication Tools

Participants’ opinions on virtual audits, virtual meeting.

*“In terms of our meetings and our regional antimicrobial pharmacist meeting… all being done by Zoom since the start of the pandemic. Our audit and QI work… was limited because of problems with movement around the COVID and non-COVID wards, but we have restarted some of that. And then training and education obviously stopped at the start of the pandemic but then we moved to use remote. So, a lot of our training via Zoom or we do small sessions if they’re workshops and where we are able to socially distance.”*

(PI12)

### 6.3. Relaxed Bureaucratic Procedure

All the participants were encouraging flexible or remote working, which assisted them in working efficiently at different working sites or work from home.

*“But there are opportunities that we can improve AMS by remote working. So, we were able to get these lists prior to the pandemic, but we can get a list of patients who are on antimicrobials, and we can review them remotely, and we can make recommendations remotely. So, it means that we have the potential to see a wider number of patients, … more wards, and it doesn’t matter which side of the city we were working out, we can still review patients in most cases remotely. And so, I think they are potentially some positive impacts on AMS but ones that we haven’t fully explored yet.”*

(PI13)

### 6.4. Advancement in Technology

Participants were enthusiastic about introducing e-prescribing and how it would help to perform AMS activities.

*“The other big change for us is that we have been introduced to electronic prescribing about six or seven or eight months ago and that is a big help to AMS activities because it just refines information in one place. In pre-electronic prescribing, we had to look at drug charts and so that meant we could not do remote work. We had to find drug charts that were never there, we were always lost. So yes, in the IT developments have really helped us a lot.”*

(PI02)

## 7. Discussion

This study offers the perceptions of AMS team members in secondary health care settings advocating the importance of antimicrobial stewardship activities during the pandemic. Participants reported experiences of various AMS activities implemented before the pandemic and the challenges AMS teams encountered during the pandemic, including a lack of proper guidance from public authorities. To overview stewardship activities, various national groups are responsible for creating different strategies and implementation tools, which include the Antimicrobial Stewardship subgroup (ASG) of the Advisory Committee on Antimicrobial Resistance and Healthcare-Associated Infection (ARHAI) and English Surveillance Program for Antimicrobial Utilization and Resistance (ESPAUR) in England, Scottish Antimicrobial Prescribing Group (SAPG), Welsh Antimicrobial Resistance Program Surveillance Unit (WARP-SU) and Antimicrobial Resistance Action Committee (ARAC) in Northern Ireland [38]. A quantitative survey-based study was also published assessing the impact of the COVID-19 pandemic on AMS activities, illustrating the negative impact in the secondary healthcare field [29]. A semi-qualitative study conducted in the primary care setting described a decline in the antibiotic prescribing threshold during initial phase of pandemic for respiratory tract infections [39]. In our study, participants identified that AMS activities including routine AMS ward rounds, education and training, auditing, and updating local or national guidelines were disrupted during the initial phase of the pandemic. This resulting lack of AMS involvement and engagement occurred due to staff pressure. Core AMS activities such as, AMR surveillance, auditing and monitoring may have massive impact on antimicrobial resistance and various studies highlighted the impact of the pandemic on increased AMC and reduced negligence on AMR surveillance [9,40].

Various telecommunication tools, flexible and remote working, and hybrid working were introduced to resume healthcare activities and study participants were also in favor to resume AMS activities remotely. A hybrid working environment, i.e., remote or flexible working was the most ubiquitous approach to resuming AMS activities as the new normal, following the COVID-19 pandemic. In the NHS workforce, remote working has enormous implications for averting staff vacancies and reducing workload [41]. A qualitative primary care studies on transforming of primary care services were published including one study from gathering data from eight European country included UK focusing on challenges faced by primary care professionals delivering health care services [42]. Another mixed-method study also emphasized on the constraint and limitation of the remote working [43], whereas our participants perceived shifting to remote working as beneficial for secondary care services to resume AMS activities, however it was still a challenge to resume as pre-pandemic. Participants acknowledged flexible and remote working even though they indicated concerned about building relationships with their other colleagues. In our opinion, it may also impact the point of patient care with virtual consultation and interactions for better patient satisfaction. In community services, it had been found that online consultations were causing irrational antimicrobial prescribing [39].

Despite the fact COVID-19 is a viral disease, most of the participants mentioned about irrational antibacterial prescribing whilst initiating pervasive procalcitonin (PCT) testing had an enormous impact on antimicrobial consumption and a helping tool for stewardship activities [37,44,45]. A multicentre study in the UK, emphasised the importance of procalcitonin to curb the irrational use of antibiotics [46]. Besides, irrational antibacterial consumption, widespread antivirals used in COVID-19 patients remained common. Various antivirals have been used since the inception of the COVID-19 pandemic, however further studies are required to evaluate the effectiveness of antivirals [47] but only a few participants were indicating this issue and started to review antiviral as stewardship activity in their facility.

In contrast with the new AMS strategies adopted to resume AMS activities; e-prescribing (ePMA/EPR) was assumed to be the most valuable in those facilities that already implemented it. In our study, consultant microbiologists and lead AMS pharmacists highlighted the importance of electronic systems and referred as the challenging to AMS activities in the absence of an electronic system during pandemic. COVID-19 pandemic has acknowledged the significance of e-prescribing and numerous NHS trusts and services work on initiating ePMA/EPR and improving the implementation process of health care information system [48]. Furthermore, the primary challenge during the pandemic was the lack of resources, staff vacancies, and the redeployment of designated staff to other duties. We also found that most participants felt that “AMS is not the priority”; everyone was anxious about COVID-19 disease, infection control and prevention to reduce the transmission as well as treating patients with COVID-19 disease. The lack of guidance, unclear information, and delays in updating national or local AMS guidelines from public health authorities during the pandemic was also overlooked [49]. However, various clinical trials were conducted, such as RECOVERY trails, which concluded the use of dexamethasone and no antimicrobial necessary for COVID-19 patients [50]. Most participants gave positive views on reducing bureaucracy during COVID-19, enabling frontline staff to deliver changes to AMS activities for improvement of patient care [51].

Moreover, in our study, participants mentioned that AMS must be everyone’s responsibility although the conventional stakeholders including senior leadership, medical microbiologists, infectious disease consultants, AMS pharmacists, AMS nurses and primary teams are the core members, however each person involved in antimicrobial prescribing must be accountable.

### 7.1. Strength and Limitation

Our study focused on key AMS challenges during the pandemic in secondary healthcare services. Our study identified mixed views of participants on AMS activities in UK hospitals and the summary of the positive and negative impacts of the COVID-19 pandemic on hospital AMS activities is presented in Tables S1 and S2 (see the supplementary). The key strength of our study was to identify various AMS implemented activities in secondary health care sector. In contrast, several studies from primary health care revealed changes in AMC and the working environment, i.e., remote working. Our study identified training and teaching was the most abundant AMS activity during pandemic. A further strength of our study was the identification of reduced auditing, monitoring and quality improvement activities like CQUINs. Hybrid working environments facilitate antimicrobial stewardship activities but in participants’ views, this may possibly be challenging to resume as post-pandemic. The study has some limitations. Firstly, participant recruitment was low, which may have been due to requests being sent during a COVID-19 surge in hospitals, however, saturation of themes was reached with the number of participants. After interviewing 17 participants, data saturation was observed as no new themes have emerged, and thus there was no need to recruit additional participants. This is in line with a recently published systematic review of 23 articles of which the authors highlighted that 9–17 interviews are sufficient to reach saturation [52]. Secondly, the representation of AMS specialists among the included participants was not homogenous. However, this was expected due to clinical pressures relating to COVID-19 and the unavailability of other team members. Despite this, a good mix of pharmacy and medical staff was recruited from all four countries in the United Kingdom. Moreover, our interview questions along with the different specialties that were interviewed, explored the challenges the whole AMS team members have faced, so including a non-homogeneous group may have helped to explore challenges that were experienced differently amongst the individual team members.

### 7.2. Future Research Recommendation

We suggest reviewing antivirals as an audit and quality improvement initiative to evaluate the effectiveness of antiviral therapy and reduce irrational use. We propose further research to assess the relationship of suspended AMS activities, antimicrobial consumption trends and antimicrobial resistance during pandemic. Furthermore, our study revealed positive impact of remote or flexible working on AMS implementation and activities and how this new hybrid working influences AMS teams and building relationships with colleagues should be evaluated.

## 8. Materials and Methods

This qualitative study followed standards for reporting qualitative [53]. A phenomenological approach using a qualitative study design was employed using in-depth interviews to explore participants’ experiences and perceptions about implemented antimicrobial stewardship activities or strategies before and during the COVID-19 pandemic. The study also investigated how the COVID-19 pandemic affected routine AMS activities or strategies and aided the development of new AMS strategies.

### 8.1. Inclusion and Exclusion Criteria

Study participants were practising healthcare professionals including pharmacists, infectious specialists, and others. All recruited participants must have been part of an AMS team during the pandemic and were provided with the study protocol, participant information sheet, and the consent form as shown in the Supplementary Material (Supplementary Materials File S1).

### 8.2. Sampling Strategy and Recruitment

Convenience sampling was used to recruit the target study population. Participants were recruited by sending an open invitation to members of various clinical forums, including the British Infection Association (BIA, E-list), United Kingdom Clinical Pharmacy Association (UKCPA)—Pharmacy Infection Network, Yorkshire and Humber Antimicrobial Pharmacy Group, Scottish Antimicrobial Prescribing Group (SAPG) and antimicrobial pharmacist WhatsApp group (some invitees were members of more than one group). Participants were contacted to arrange an interview, and written consent and participant characteristics data were collected before each interview and verbal informed consent was also taken before the interview. A total of 1782 invites were sent to medical microbiologists, infectious disease consultants, pharmacists, and nurses; 25 participants responded to the invitation by email, and six participants dropped out either because of clinical duties or lack of time. As a result, 19 participants from the UK were reconfirmed for participation, and two were chosen for the pilot interview.

### 8.3. The Interview

Semi-structured interviews were conducted through Microsoft Teams and recorded by the research team member (SK) using a semi-structured guide. An interview guide was developed based on the author’s research expertise and other studies conducted on related topics [29,39] (Supplementary Material: Supplementary Materials File S1). The first two pilot interviews were conducted to test and validate the interview questions and the recording process. The study participants were not familiarized with the researcher before the interview questions to explore perceptions and views about AMS activities before the pandemic and how they were impacted during the pandemic.

### 8.4. Ethics Approval

Ethical approval was obtained from the School of Applied Science, University of Huddersfield (*Reference No: SAS-SRIEC -11.1.22-1*). Before we started interviewing the participants, verbal consent was obtained from all participants. Confidentiality and privacy were guaranteed during the study. All collected data were stored anonymously and saved on a secure University drive; only the research team had access to the data.

### 8.5. Data Processing and Analysis

The audio-recorded interviews were transcribed verbatim, notes were made during the interviews, and participants were asked for any clarification needed. Interview transcripts were not returned to participants. Each transcript was reviewed line by line and then verified. The transcripts were then thematically analysed using the framework technique [54]. The identifying themes and new themes were added as they emerged during the subsequent inductive thematic analysis [54,55]. After interviewing 17 participants, data saturation was observed, and no new participants were recruited.

All transcripts were uploaded to qualitative data management software (NVivo, v.12, QRS International). Two researchers (SK and AAS) analysed the initial work thematically. Five transcripts were inductively coded and then compared for integration of codes. A coding framework was developed, and the higher-level categories and themes were identified after agreement, and using the defined coding framework, remaining transcripts were coded (17 coded by SK and five coded by AAS). SK and AAS independently led the thematic analysis, and consensuses with the coding frame were agreed with input from MB and MAA. All coding was assessed for uniformity, and a team-based approach (“researcher triangulation”) was adopted to ensure consistency of the analysis.

## 9. Conclusions

Our qualitative study concluded that most of the antimicrobial stewardship activities were completely withheld during the initial phase mainly in 2020. However, after the introduction of the new delivery system in healthcare, a variety of activities were resumed and re-initiated including virtual meetings, virtual ward rounds, online audits, e-learning and educational activities. Furthermore, this hybrid-working environment supports healthcare professionals to work remotely efficiently in their perceptive and a good initiative during the pandemic. Though our finding suggested, this hybrid-working will not be valuable to resume all AMS activities as pre-pandemic, which is still a matter of concern. We recommended local and national strategies must be reinforced and should be implemented, as AMR thought to be the major global threat.

## Figures and Tables

**Table 1:** Participants’ characteristics.

Participants Characteristics	Number of Participants (n)
Age	
30–39 years	3
40–49 years	12
50–59 years	2
Current Role in AMS	
AMS Pharmacist	9
Infectious Disease and Medical Microbiologist (IDM)	2
Infectious Disease Consultant (ID)	1
Medical Microbiologist Consultant	5
Gender	
Female	13
Male	4
How many years of affiliation	
2–5 years	5
6–9 years	4
Less than 2 years	3
More than 10 years	5
Location	
England	11
Northern Ireland	3
Scotland	1
Wales	2

**Table 2:** THEME ONE: AMS activities or strategies before and during the pandemic.

Subtheme	Code	Illustrative Quotes (Participant Number and Role)
**AMS** **WARD** **ROUNDS**	Handling of IV antibiotics	“… we did a small an activity of antimicrobial stewardship ward round that gave us this information that we then used and then we focused creating a specific IVOST a guideline that was initially directed at the surgical teams, which really simplified the IVOST process for them what to consider which agents to use and that’s that seemed to be ineffective locally.” (PI01, IDM) “… give all the tools to the prescribing teams to be able to switch from IV to oral antibiotics as soon as they can, and we are still in this kind of work, and we are hoping to do a bit kind of campaign around increasing the profile of IV to oral switches as well to follow on with that work.” (PI11, PH)
	MDT ward rounds	“The thing that was probably impacted the most was the micro ward rounds because the microbiologists at that time we only had two, and they were so caught up in COVID planning that they didn’t have the time to do a ward round… So those ward rounds were impacted the most.” (PI04, PH) “… we had a twice weekly antibiotic ward rounds which was attended by the duty microbiology consultant registrar, if we had a registrar at the time, the antimicrobial pharmacist and sometimes an infection control nurse… that ward rounds would target patients with significant culture results.” (PI17, MB)
	Challenges in building relationships	“The ward rounds, I think are extremely effective. So, I think you know when they see you regularly, they build up a relationship with you and they trust your advice and they know that you are there for the patient as well. And you can explain the rationale for why, patient doesn’t need antibiotics or doesn’t need that particular antibiotic and… can prevent that whole week of unnecessary broad-spectrum antibiotics, I think those are the most effective things.” (PI05, MB) I think the main thing that hindered was there were previously ward visits by microbiologists and ID where they would go physically see the patient and obviously, they all had to stop due to the pandemic…” (PI13, PH)
	Virtual ward rounds	“… we now do instead is virtual antibiotic ward rounds. So, we do that twice a week as well as the antibiotic pharmacist creates a list of patients whom he is identified as potentially being on inappropriate antibiotics. … So, a lot of that though is still done from our office rather than going out on the wards.” (PI17, MB) “… really big positives from COVID have been the use of Microsoft Teams, we can now do Ward rounds remotely that wouldn’t have been possible before… I can check who’s on antibiotics. I can speak to a pharmacist to somewhere else, and I can speak to a haematologist to somewhere else.” (PI02, IDM)
**AUDITING** **AND** **QUALITY** **IMPROVEMENT** **ACTIVITIES**	Quality improvement activities	“Big strategy is audit and quality improvement projects where we see what’s happening in the trust and see if there are ways that we can try and improve stewardship through intervention and then follow up on that.” (PI12) “Our audit and QI activity and a quality improvement activity because a lot of it was halted initially and although we are starting to bring that back and we do have a lot of small audits. It has been difficult to get that back on track to where we were.” (PI12, PH).
	CQUIN activities	we were working very closely with the CQUIN to reduce AMR and the national contract. So, reducing our antimicrobial footprint…” (PI07, PH) “we do the quarterly antimicrobial prescribing audits… We did a lot of work on the CQUIN audit and try to improve things like diagnosis and management of UTIs.” (PI05, MB)
	HARP	“And that’s kind of where we were pre- COVID, the Scottish Antimicrobial prescribing group had just launched the HARP (Hospital Antibiotic Review Program) toolkit, and we were just starting to look at how we would use that as a tool and where we would employ it.” (PI01)
	Start-smart-then focus	“The biggest thing is to start-smart-then-focus audits. So, what the success from our side is that we have trained all of the junior doctors, it is part of their rotation. We have it agreed that it’s a mandated audit, it’s what we call a Tier 2 audit in the hospital. That means it’s reported all the way up to the board. So that is the biggest thing that we have that’s been successful.” (PI08).
	Procalcitonin testing	“I have forgotten about that only one that we introduced that more widely across the hospitals and the use of procalcitonin starts for our auditing.” (PI08, PH) “we had huge increase in doxycycline use later on during the second phase… and that was at a time when we had introduced procalcitonin testing… was effective in reducing doxycycline use down to a normal level, and now procalcitonin is used routinely in patients with COVID…” (PI04, PH)
	Antibiotic Review Kit (ARK) trial	“Before the COVID pandemic… there was the ARK (Antibiotic Review Kit) study… where we had automatic stop dates of antibiotic prescriptions after 72 h. So, if you wanted to prescribe more than 72 h of antibiotics, we had to… re-prescribe it on the Kardex and that influenced and increased our IV to oral switch rate that was again before the COVID pandemic.” (PI04, PH) “… the antibiotic review kit and this was part of a wide research project that was done across a lot of the UK sites, and it’s already worked out of it or not, but it’s around behaviour change around antibiotic prescribing, what it meant in practice for us so that we amended our prescribing chart so that antibiotic prescriptions are only lasts for three days in the first instance. The prescription will then stop unless the prescription is actively rewritten. So, this is something we did as a research project to start within medicine…” (PI11-WL-PH)
	(Online) audit process	“we have started using an online audit tool for stewardship, so this is something that we have received funding from a company, and we have made a database of all of our well patients for a time period… So, I mean, not just the MDT, but we have also got an online database of patients who were collecting and who we are presenting data from and hoping to publish our experience of AMS as well…” (PI02, IDM). “Our audit and QI work did an initially, it was limited because of problems with movement around the COVID and non-COVID wards, but we have restarted some of that. And then training and education obviously stopped at the start of the pandemic but then we moved to use in remote.” (PI12, PH) “So, across four sites we have undertaken audit every month, and the results of those audits would have been feedback and monthly reports, but also, they would have been feedback at the monthly medical mortality and morbidity meetings…” (PI04, PH).
**EDUCATION** **AND** **TRAINING**	Commitment and engagement	“We would have undertaken a range of educational activities with pharmacists, medical staff, and nursing staff, and we had monthly F1 teaching sessions where we would just talk about current issues and antibiotic prescribing, and then in certain months, we might have had a more focused teaching session… So, we are very good at engaging monthly with medical, nursing, and less pharmacy staff.” (PI04, PH) “Small teaching sessions, in groups of between five and eight people looking at case studies specifically with real life patients and having open discussions about strategy for treatment and improving their knowledge base and the knowledge base round antimicrobials and the spectrum of activity around these antimicrobials and why we use them, and they were able to then take, these factors then their day-to day practices.” (P116, PH)
	Innovation in education and training	“I say that teaching a lot of it stopped, but it did present some additional options for teaching. So, for example, I was asked to give a teaching session to the acute medicine doctors about the use of procalcitonin testing. So yeah, some teaching was replaced by teaching that wouldn’t have otherwise the patterns under normal circumstances. (PI17) “in conjunction with the clinical team, we discussed the antibiotics at the patient’s bedside. We encouraged junior doctors, other prescribers, ward nurses, etc. to come on those ward runs with us so that it was an educational and informative experience rather than just us coming in and writing the notes and then running away.” (PI06, MB) “one of the strategies we were looking at employing we have and on to antimicrobial specialist nurse in our organization and we were looking at nursing education and how we could empower our nursing teams to have more awareness around antimicrobial stewardship and be prompting review of antibiotics.” (PI01, IDM)
	Online education and training	“That’s the first year, there was a definite huge reduction in teaching that we could deliver. There is almost none… The second year’s teaching has resumed at the same frequency, but it’s been delivered largely remotely… So, I think that’s been a hindrance, and I look forward to the day that we can start doing that sort of thing in person again. I think that would be much improved.” (PI05) “education has been a bit of an issue. We have managed to do some online training and I was lucky to be part of some of the training dates that we were be able to provide during this pandemic to support other micro pharmacist.” (PI07, PH)
**ANTIMICROBIAL** **GUIDELINES**	Access to the guideline	“The guidelines antimicrobial guidelines are key part to it, we have got very comprehensive guidelines available in both on and off, but and a website as well. And so, their key in directing antibiotic prescribing within the hospital and we also do a lot of prescribing surveillance.” (PI11-WL-PH) “So before so we have done quite a few things, we set up our micro guide few years before to what guidelines are now sit on our micro-guide and all that had been running and one of the biggest pieces of work, we do is obviously keeping it up to date and maintaining it so that is one of our one of our good projects.” (PI07-EL-PH)
	Obstacle in adopting update guidelines	“when the pandemic started there were an awful lot of guidelines first published under the Public Health England badge and then subsequently under NICE guidance and some of these were published very short notice normally on Friday afternoon. And they did not always make it easy to try and keep your existing stewardship activities going…” (PI06-NI-MB) “We know we have seen prescribing get out of hand really and it took a long time to get on top of this to restart activities, to write new guidelines, you know, particularly thinking about colleagues who work in ICU who was struggling with the explosion in not just antibacterial but antifungal prescriptions.” (PI02-EL-IDM)
	Antibiotic policy	“We managed to change some longstanding antibacterial prophylaxis policies in September 2019 and so, we were definitely active in the AMS, and we were doing things and getting some results before the COVID pandemic.” (PI02) “And the other thing we do is, we have a micro guide electronic app… with all our antimicrobials policy on it and what we do is twice a year we do a snapshot point prevalence audit of the compliance with our micro guide… so we can see… how well everyone sticking to policy and see if there are any particular teams on the areas that… we need to work in.” (PI15)
**OPAT** **SERVICES**	Negative impact of pandemic	“I am sure the lack of availability we have had a big impact on our OPAT service. We had to pause our OPAT service during the pandemic again because we’re a smallish centre and we didn’t have the resource as well as running a COVID ward, we are now realizing how negative that was as we start restarted the service, and we are seeing patients who should have been treated appropriately, sometimes with longer courses of antibiotics and in fact, they were given up for specialist infections who… did not have as good outcomes as they might have had if we had a functioning well OPAT service and that access to specialist advice.” (PI01, IDM) “…there were a lot of changes based on difficulties with staffing, in terms of nursing, they pulled (to) cover the nursing homes and managed COVID patients. But then that has resolved but… structurally they changed and for some patients complex or (oral) regimes were used instead of IV if that was necessary due to capacity.” (PI12)
	OPAT services	“… during this pandemic we have actually introduced OPAT into the organization where we actually treat patients in their own homes after we have for long term infections and the feedback, we have got from patients is they find this an amazing thing to be treated in their home. They are not hospitalized for weeks, and they have all their home comforts at hand and also, they are not exposed to hospital acquired infections as they would previously be if they were being treated in hospital.” (PI16, PH)
	MDT OPAT services	“I guess setting up the OPAT MDT because that was done relatively recently there was in the last three or four years and prior to that we hadn’t had particularly good oversight of patients who were on OPAT in terms of monitoring the blood tests and it was really left to the consultant of care… So, introducing that weekly MDT meant that we could flag up any abnormalities in blood results to the consultant of care…” (PI17, MB)

**Table 3:** THEME TWO: Challenges to implementing AMS activities before and during the pandemic.

Subtheme	Code	Illustrative Quotes (Participant Number and Role)
**INTITUTIONAL** **CHALLENGES**	Organizational priorities	“we didn’t have the capacity within the antimicrobial stewardship team, which is I am sure that most hospitals, quite a small number of people to follow up in those activities because they were infectious, a lot of our AMT come from our infectious diseases department and our microbiology department or infection control, all of whom were heavily involved in the COVID response…” (PI01, IDM) “… I don’t think it was an organizational priority here. I didn’t feel that it wasn’t something I was ever asked really to talk about report on during the pandemic. There is so much focus on the acute issues of COVID dealing with COVID patients. The bed based, the demands on the system staffing crisis. Yes, I don’t think, it fell in our hospital’s priorities or on the radar of the wider hospital at all actually.” (PI01, IDM) “… AMS is never high on the agenda of- It’s not high. It doesn’t feel like it’s high on the agenda of our trust. So mainly is our microbiology and infection department that pushes AMS activities. So, we do sometimes think that if it wasn’t for our department, there wouldn’t really be any AMS, but I think that’s the same everywhere could have imagine.” (PI02-EL-IDM)
	Lack of support	“we know we don’t have electronic prescribing here, it’s still written on Kardex, and some words have notes, some words of electronic notes. So, we did try and move to paperless at the start of COVID, but that was just more for pharmaceutical care plans for patients with complex infection and we are able to look up some of the notes just online, but we still need to physically go to the ward look at the Kardex.” (PI12, PH) “… You know without the right data, without the right business intelligence to say you know this is where prescribing is highest, or this is how we can change things on the electronic prescribing to make improvements in how we prescribe antibiotics. You know, we might have these great ideas, but until we can get that sort of Technical Support to make it happen, that’s the major barrier.” (PI05, MB)
**HIGH** **ANTIMICROBIAL** **CONSUMPTION**	Irrational antimicrobial prescribing	“it was very difficult and remains very difficult to tell the difference between a viral pneumonitis and a bacterial pneumonia or just a bacterial pneumonitis itself. It is the result of that people were prescribing drugs like co-amoxiclav or Pip/tazo where they would normally have prescribed a narrow spectrum drugs like amoxicillin or indeed just not given an antibiotic and waited to see what happened. It became very much a start-up broad spectrum and worry about it later or a give it a short course and stop it. So, it was very difficult to try to stop people panicking.” (PI06, MB) “so at the start of the pandemic, I would say no because we had quite an irrational use of antibiotics, it was given to everybody. However, as time moved on, we started to get the support of clinicians outside of the core antimicrobial stewardship team, such as the intensivist, such as the medical doctors who were covering, who ended up on the COVID rotations and they then started to highlight the use of inappropriate prescribing and that we need to curb the amount of antibiotics were using.” (PI08-WL-PH)
	Misuse of antibiotics	“… I think there was also a lot of misinformation at the beginning of the COVID pandemic. So, antibiotics which were part of trials for example Azithromycin, I think was in the recovery trial was used off label widely. So, there was a lot of chaos and rumour and, people just prescribing something that they have heard off on the news and so ferment for all those many reasons. I think antibiotics pretty much wherever antibacterial wherever you look have been misused during the COVID pandemic.” (PI02-EL-IDM)
**INDIVIDUAL** **LEVEL** **CHALLENGES**	Commitment and engagement	“… I think we all take responsibility of ensuring antimicrobials used appropriately quite strongly. So, I do think we committed to helping ensure that and you know the ward rounds are highly valued. So yeah, I do believe that we were committed to implementing the strategies.” (PI10-EL-PH) “I think the first year there was a huge reduction in the number of meetings, and everyone just sort of to make major changes in lab testing and clinical pathways in the trust and infection control protocols. So yes, reducing meeting burden that should have been a good thing, but obviously there was potentially less focus on AMS at that point.” (PI05, MB)
	Lack of time and support	“… AMS ward rounds have probably been impacted the most. For various reasons, the lack of time, the lack of physical space, sometimes the lack of priority. It’s not seen as very important in the middle of a national pandemic because a lot of people see AMS is restricting antimicrobials…” (PI02, IDM)
**COLLABORATIVE** **WORKING**	Relationship with colleagues	“I think the key to running AMS programs in any department a relationship building. So, you know, finding interested champions of AMS within medical department, haematology department or ICU department and it’s down to us as microbiologist or infection doctors to cultivate these relationships. And only if we have these relationships with the department that we want to change is there any hope of changing.” (PI02, IDM) “I think certainly is an AMT between myself and antimicrobial stewardship nurse and antimicrobial pharmacist, we were certainly very committed to. But I think we were constrained by time and availability. We have quite a lot of variation over the across the organization, we have some individuals within the organization who are highly committed and have led working in their own area around about AMS…” (PI01, IDM)
	Involvement of clinical team	“Obviously I would prefer the clinical team to review the antibiotics daily and stop it accordingly in their ward round because obviously there is only two consultants working in this hospital. And yes, I do get the support because if I stop the antibiotics myself and most of the time, they do kind of accept that…” (PI09, MB)

**Table 4:** THEME THREE: Information from public authorities on AMS during pandemic.

Subtheme	Code	Illustrative Quotes (Participant Number and Role)
**ENGAGEMENT** **AND** **CLARITY**	Lack of clarity	“… I am you know, the operational lead for COVID infection control. I don’t particularly remember seeing anything useful from public authorities about implementing antimicrobial stewardship activities during COVID. I mean, we made a decision to introduce procalcitonin testing to help guide when it would be useful to use antibiotics or not…” (PI15) “… Sometimes we felt the information we got was very short notice that we had very small timelines to turn things around, especially with some of the new treatments that came out that we had to you know, implement them getting into daily practice within, you know, overnight pretty much and we found that quite challenging.” (PI07, PH)
	Lack of engagement	“I think that the health boards drive the agenda rather than public health… doing so. And often, its actions and initiatives that we have done back in the health boards that we feed up to our own delivery board that then changes national strategy. I don’t believe that public health has a good buy-in, I don’t believe that they provide good data, and I don’t believe that they give us good information on prescribing rates or anything out from that side at all…” (PI08)
**SUPPORT** **AND** **GUIDANCE**	Lack of guidance	“I don’t think public health authorities helping in anyway because I don’t think they have concentrated on this aspect at all. I don’t think I would have to say public health authorities throughout the pandemic, I think they haven’t been straight, rationally in many of their advice I have to say… I don’t know, I think in terms of antibiotic stewardship, public health authorities probably didn’t see it as their role to be honest.” (PI14, MB)
	Lack of information	“we didn’t receive any information from the from our local public health agency on antibiotic stewardship during COVID… And each trust has taken a different approach and we’ve all kind of just gone and done our own thing on what we think, and you know, we should be doing. So, that’s my opinion. I don’t think we had any guidance from public health agency on what we should be doing regarding after antimicrobial stewardship.” (PI04, PH) “I don’t really remember receiving very much information about implementing activities during the pandemic. I think a lot of it just stopped because people didn’t have the time to give to that. I mean, I know there were eventually some NICE guidelines published on antibiotic prescribing, but I felt they probably came a little bit late and were a bit vague and actually, didn’t reflect what we were doing. And so, I didn’t find what came from the national sources particularly helpful.” (PI17, MB)

**Table 5:** THEME FOUR: New AMS activities/strategies adopted during the pandemic.

Subtheme	Code	Illustrative Quotes (Participant Number and Role)
**DIAGNOSTIC** **BIOMARKER**	Procalcitonin testing	“it is not a perfect test, but generally procalcitonin is felt to be more of an accurate marker for bacterial infection than viral. So, if you have a patient who has come in with a severe respiratory infection who’s tested COVID positive where the clinical team might want to prescribe a broad-spectrum antibiotic, we have asked them to do a procalcitonin and if the procalcitonin is normal to hold off on to the antimicrobial and actually just treat the COVID.” (PI06, MB)
**COMMUNICATION** **TOOLS**	Virtual meetings	“We have brought in a few changes to that where it might be more telephone based or are trust user zoom rather than teams, so it might be a zoom meeting with the teams on the ward, we have not got back to a point yet where our enhanced stewardship rounds are back running, except for the case of the intensive care round and the neonatal round. So, we are doing those, but we are doing those virtually through a video link.” (PI06, MB)
**RELAXED** **BUREAUCRATIC** **PROCEDURE**	Impact on bureaucracy	“And I certainly think that reduced bureaucracy has been beneficial, and we are in the process of rewriting our guidelines. And I think there is much more of an acceptance that guidelines are dynamic and that they are ideally, I think should be short and focused. And go through a relatively simple approval process rather than our previous approach, which was to have a very lengthy and exhaustive antimicrobial guidance policy.” (PI01, IDM) “I think that bureaucracy is probably stayed the same, but we have also tried to be a lot more pragmatic about how we get things through. So, we have had a guideline for COVID and normally a guideline can take between three and six months from start end to come to be published if it’s a new one. We managed to do it with a rapid approval process, and it was up very, very quickly.” (PI13, PH)
**ADVANCEMENT** **IN** **TECHNOLOGY**	Virtual ward rounds	“I think the development of virtual ward rounds has worked well where we brought it together for enhanced stewardship service. I think investing in technology and trying to enable clinicians to take ownership of their own stewardship see it as they would any other.” (PI06, MB)
	e-prescribing	“I am very keen on using anything where technology can assist us, whether it’s app-based technology or easy to access guidance or on the Internet. So, I think for us that would be a strategy that would help, because we have tended to have our guidance hosted within formulary pages and other areas where it’s actually quite difficult to access unless you know what you’re looking for.” (PI01, IDM) “I think the integration of electronic prescribing is definitely helped in certain aspects and has may be hindered us in other aspects, particularly review data as a troublesome topic. But I think what has worked well so in electronic prescribing is the order sets that we use, which aid prescribers in prescribing the correct dose frequency for a given drug, if there is a particularly complex regime and that has reduced errors in prescribing and administration from that perspective.” (PI03-EL-PH)
	Automatic antibiotic stop	“… I talked about the antibiotic review kit (ARK) and the chart with a three day stop on it… we did support kind of that implementation with dashboards that prescribers could see easily who’s on antibiotics and at what stage antibiotic prescription there are at and to change the pull a focus on antibiotic prescribing and to minimize the risk of a prescription been stopped inadvertently as well.” (PI11-WL-PH) “during the pandemic, we strengthened for example, COVID antiviral protocol as well, we built automatic protocol in our prescribing system, so doctor could simply tick a box, and everything get transcribed.” (PI14-EL-MB)

## Data Availability

Relevant information are contained within the paper and the Supplementary Material.

## Supplementary Materials

The following supporting information can be downloaded at: https://www.mdpi.com/article/10.3390/antibiotics11111600/s1, Interview Guide: 1.1 Participant information sheet; 1.2 Consent Form; 1.3 Interview guide; Abbreviations; Table S1: The negative impact on Antimicrobial Stewardship activities during the COVID-19 pandemic; Table S2: The positive impact of the COVID-19 pandemic on antimicrobial stewardship (AMS) activities.
