# Supplementary material for: COVID-19 Mixed Impact on Hospital Antimicrobial Stewardship Activities: A Qualitative Study in UK-Based Hospitals
Source: Antibiotics (Basel). 2022 Nov 11;11(11):1600. doi: 10.3390/antibiotics11111600 (PMC9686587; doi:10.3390/antibiotics11111600)
Supplement: Supplementary file 1 [file antibiotics-11-01600-s001.zip › antibiotics-1979033-supplementary.pdf]

Supplementary Materials: Interview Guide (Supplementary Materials File S1)

1.1. Participant information sheet

The information provided below will help you to understand why we are doing this study and what it is about. Please read provided information below and do not hesitate to ask for clarifications or more information. We would like to take this opportunity to thank you for taking the time to read this.

What is the purpose of the project?

Antimicrobial stewardship (AMS) programmes were established to inform rational use of antibiotic prescribing, prevent the emergence of antimicrobial resistance, improve patient healthcare outcomes, and reduce incurred healthcare costs. These programmes were highly challenged during the COVID-19 pandemic. The nature and impact of AMS initiatives during the COVID-19 pandemic remain unclear.

Aims and objectives:

This study aims to investigate the impact of COVID-19 on the delivery of AMS activities and antibiotic prescribing in UK hospitals. This study will also explore what new strategies were implemented and members of AMS teams' perceptions on how effective they were in optimising the antimicrobial use, and whether we can adopt these strategies in hospitals during ongoing COVID-19 or any future pandemic.

Specific objectives:

-To explore what AMS strategies were employed in hospitals before the COVID-19 and the opinions of members of AMS teams about the effectiveness of these strategies.

- To explore the structural changes that occurred because of COVID-19 (e.g., reduced meeting burden, reduced bureaucracy, use of remote and flexible working, etc.) and their impact on AMS activities in hospitals.

- To discuss the barriers and facilitators in providing AMS services during the COVID-19 pandemic

-To explore any new or different activities adopted in hospitals to overcome the challenges in providing AMS services and explore the opinions of relevant AMS clinical staff about the effectiveness of these activities.

Why am I doing the project?

This study is being conducted to help understand the impact of COVID-19 on the delivery of AMS activities and antibiotic prescribing in UK hospitals. The outcomes of this study will inform what new strategies were implemented and members of AMS teams' perceptions of how effective they were in optimising the antimicrobial use, and whether we can adopt these strategies in hospitals during ongoing COVID-19 or any future pandemic.

Do I have to take part?

Your participation in this study is completely voluntary and there is no obligation to take part in it. Nevertheless, your participation is essential for this study to help with achieving the aims and objectives mentioned earlier, therefore, we would encourage you to participate.

What do I have to do if I agree to take part?

You will be invited to take part in an interview that should take no more than 30-45 minutes of your time. The interview will be conducted on teams (only audio). Discussions taking place during the interviews will be recorded and then transcribed by the project coordinator's team. Following transcription, the recordings will be deleted. Only the research team will have access to this data. All collected data will be anonymous with no participants' identifiers.

What are the advantages of taking part?

Your participation in this study will help with achieving the study's aims and objectives. Taking part in this study will help with determining AMS activities that were effective in optimising the antimicrobial use and whether we can adopt these activities in hospitals during ongoing COVID-19 or any future pandemic. It is anticipated that the overall results of this study will inform the development of policies and activities to improve antimicrobial stewardship practice in hospitals.

Are there any disadvantages to taking part?

Other than the time required, there should be no anticipated disadvantages to your participation. At any stage during the process, you can ask for further information or raise concerns directly to the study team (contact information provided below).

Will all my details be kept confidential?

All information that is collected will be stored on computers within the University of Huddersfield and using OneDrive. Discussions taking place during the interviews will be recorded and then transcribed by the project coordinator's team. Following transcription, the recordings will be deleted. Only the research team will have access to this data. All collected data will be anonymous with no participants' identifiers. It will not be possible to link any particular comment to any participant.

What happens when the study ends?

The interview transcripts will be analysed and will be written up for a manuscript for publication. It is anticipated that the overall results of this study will inform the development of policies and activities to improve antimicrobial stewardship practice in hospitals.

What will happen to the results of the study?

Results of the study will be shared with antimicrobial stewardship teams and will be disseminated within peer-reviewed scientific journals and conferences.

Who has reviewed and approved the study, and who can be contacted for further information?

The study was reviewed and approved by the University of Huddersfield- UK.

1.2. Consent Form

- How old are you?

[ ] 18-29 years [ ] 30-39 years [ ] 40-49 years [ ] 50-59 years [ ] 60 years and over

- What is your gender?

[ ] Female [ ] Male [ ] Prefer not to disclose

Please confirm, by marking the boxes, that you agree with the following statements:

1.	I have read and understood the information about the project above, as provided in the Participant Information Sheet.	☐
2.	I have been given the opportunity to ask questions about the project and my participation, and have received answers to any questions raised	☐
3.	I voluntarily agree to participate in this project.	☐
4.	I understand I can withdraw at any time during the interview without giving reasons and that I will not be penalised for withdrawing nor will I be questioned on why I have withdrawn.	☐
5.	The procedures regarding confidentiality have been clearly explained to me.	☐
6.	I understand that discussions taking place during the interviews will be recorded and then transcribed by the project coordinator's team. I understand that following transcription the recordings will be deleted.	☐
7.	I understand that no comments made by me during interviews discussions will be able to be linked back to me but may be anonymously quoted in subsequent reports / publications. The use of the data in research, publications, sharing and archiving has been explained to me.	☐
8.	I understand that the data will be accessed by the project coordinator's team responsible for conducting this research	☐

Name of participant, signature, and date:

1.3. Interview guide

This interview will take approx. 45minutes. Explain data confidentiality for verbal consent

- All the collected information will be stored on computers within the University of Huddersfield and using OneDrive.
- The recorded discussions taking place during the interviews will be transcribed by the project coordinator's team and will be deleted following transcription.
- All collected data will be anonymous with no participants' identifiers. It will not be possible to link any particular comment to any participant.
- Only the research team will have access to this data.

Baseline questionnaire

To begin with, we had a few questions about the participants, indicating which apply.

What is your current role in the AMS team? […………………………………………………..]

For how many years have you worked in this role?

[ ] Less than 2 years [ ] 2 – 5 years [ ] 6 – 9 years [ ] 10 years or more

Questions:

Before we start, can you please tell me what the term ‘antimicrobial stewardship’ mean to you?

Thinking about the pre-COVID-19 pandemic

1. What were the antimicrobial stewardship strategies/activities in your hospital?

2. Do you believe that these activities were effective? If so, why? If not, why not?

3. Can you share an example of a successful AMS activity you or your team have introduced?

4. Can you tell me who you believe are the important stakeholders in implementing antimicrobial stewardship strategies within your hospital? Why?

5. Do you think you (or others) felt confident that the hospital supported implementing these strategies? If so, why? If not, why not?

6. Do you think you (or others) were committed to implementing these strategies? If so, why? If not, why not?

Thinking about the current or ongoing pandemic

7. Do you think you received sufficient information from public bodies/ authorities on implementing antimicrobial stewardship activities? If so, why? If not, why not?

8. Have you come across structural changes that occurred because of COVID-19 (e.g., reduced meeting burden, reduced bureaucracy, use of remote and flexible working)? If yes, can you please elaborate on these structural changes and how these changes impact AMS

9. To what extent have these structural changes helped or hindered implementation of antimicrobial stewardship activities?

10. To what extent do you think the COVID-19 pandemic hindered the implementation of routine antimicrobial stewardship activities?

11. What are the challenges that have arisen in the use of antibiotics? Why?

12. Which AMS activity has been impacted the most? In what way?

13. Do you feel there has been any positive (or negative) impact of COVID-19 on AMS and AMC? If so, why? If not, why not?

14. Have you introduced any new AMS activities in your setting? If yes, would you please tell me new strategies/innovations you or the hospital have employed

15. Can you share an example of a successful AMS activity you or your team have introduced?

16. Do you see COVID-19 as an opportunity to emphasise the need for AMS and to curb irrational prescribing? Did you or others in the hospital feel confident in these new activities? Did you feel supported by the hospital? In what way?

17. To what extent have these strategies affected the treatment of patients with antimicrobials?

18. Based on your experience with antimicrobial stewardship during the pandemic, what other strategies can we adopt for use in hospitals

19. To what extent have clinical systems/ healthcare information systems affected your ability to perform antimicrobial stewardship activities during COVID pandemic?

20. What lessons have you learned from the ongoing pandemic, and how can this learning be helpful in future pandemics?

- Are there any other factors that we have not covered, and you think it might be important?

- Anything else you would like to add?

- Any questions?

Tables

Table S1. The negative impact on Antimicrobial Stewardship activities during the COVID-19 pandemic.

AMS ward rounds	All participants gave the impression that the most impacted AMS activity during the initial phase of the pandemic was the ward round. The decrease in footfall, redeployment of AMS staff or staff vacancies were the most contributing factors. MDT ward round, enhanced stewardship or targeted rounds were similarly affected.
Auditing And Quality Improvement	In addition to the ward rounds, auditing and monitoring were also affected resulting in irrational antibiotic prescribing. Participants all mentioned; QI (Quality Improvement) activities importantly CQUIN AMR and ARK studies were not carried forward during the pandemic. Most participant reported point prevalence survey (PPS) audits and routine audits were not conducted.
Education And Training	Several participants highlighted the lack of availability of AMS teaching to doctors and multidisciplinary teams and awareness programs specially mentioning European antimicrobial week activities during the initial phase of the pandemic, however, training sessions on COVID-19 awareness were conducted through virtual meetings.
Antimicrobial Guideline / Clinical Information	Participants had mixed opinions about updating guidelines although there were no convincing national guidelines. Several NHS trusts developed and updated guidelines on antimicrobial during the pandemic, coherent guidelines were lacking.
OPAT Services	Five participants reported due to lack of staffing, isolation, and redeployment of staff to other duties, OPAT services were withheld and restoring outpatient services back to normal was thought to be very challenging.
Public Information	Participants gave strong opinions about information on antimicrobial stewardship strategies during the pandemic, they perceived absolute absence of information. However, several clinical trials were conducted and shared outcomes.

Table S2. The positive impact of the COVID-19 pandemic on antimicrobial stewardship (AMS) activities.

Virtual meetings and ward round	Participants mentioned various online communication tools including MS teams, Zoom and WebEx that were introduced. These helped in resuming virtual ward rounds, audits, and meetings. Furthermore, teaching and training were similarly resumed as e-learning training sessions.
Procalcitonin (pct) testing	All participants reported widespread application of procalcitonin testing as a biomarker to differentiate viral and bacterial infection is beneficial to curb irrational antibacterial use.
Antiviral protocol	Only two participants highlighted antiviral AMS activities adopted during the pandemic. One participant mentioned developing a new outpatient COVID centre to review antiviral consumption.
Flexible or remote working	Participants had mixed views. Most participants also highlighted flexible and remote working eased their work and help staff work remotely work from home and cover different organisational locations. However, they were concerned about building a team, building relationships with other colleagues and direct interaction with patients and required more resources to have remote access.
e-prescribing	Most participants highlighted the importance of electronic prescribing and retrieving information promptly without visiting wards. Some participants indicated that their organisation introduced e-prescribing during the pandemic, which aiding their routine work.

Abbreviations

AMS Antimicrobial Stewardship

AMR Antimicrobial Resistance

ARAC Antimicrobial Resistance Action Committee

ARHAI Advisory Committee on Antimicrobial Resistance and Healthcare-Associated Infections

ARK Antibiotic Review Kit

ASG Antimicrobial Stewardship Subgroup

BIA British Infection Association

BMJ The British Medical Journal

CQUIN Commissioning for Quality and Innovation

DDD Defined Daily Dose

EL England

ESPAUR English Surveillance Program for Antimicrobial Utilization and Resistance

HARP Hospital Antibiotic Review Programme

HIC High-Income Countries

HCAIs Healthcare-Associated Infections

IDM Infectious Disease and Microbiology consultant

ID Infectious Disease consultant

IDSA Infectious Diseases Society of America

LMIC Low-Middle Income Countries

MB Consultant medical microbiologist

NI Northern Ireland

PI Participant interview number

PH Pharmacist

SL Scotland

SAPG Scottish Antimicrobial Prescribing Group

WARP-SU Welsh Antimicrobial Resistance Program Surveillance Unit

WL Wales

UKCPA United Kingdom Clinical Pharmacy Associatio
